# Bayesian Estimation of the Prevalence and Test Characteristics (Sensitivity and Specificity) of Two Serological Tests (RB and SAT-EDTA) for the Diagnosis of Bovine Brucellosis in Small and Medium Cattle Holders in Ecuador

**DOI:** 10.3390/microorganisms9091815

**Published:** 2021-08-26

**Authors:** Valeria Paucar, Jorge Ron-Román, Washington Benítez-Ortiz, Maritza Celi, Dirk Berkvens, Claude Saegerman, Lenin Ron-Garrido

**Affiliations:** 1 Centro Internacional del Zoonosis (CIZ), Universidad Central del Ecuador, Quito 170521, Ecuador; adriana_vale@live.com (V.P.); wbenitez@uce.edu.ec (W.B.-O.); mceli@uce.edu.ec (M.C.); 2 Research Unit of Epidemiology and Risk Analysis Applied to Veterinary Science (UREAR-ULg), Fundamental and Applied Research for Animals & Health (FARAH) Center, Faculty of Veterinary Medicine, University of Liege, 4000 Liège, Belgium; 3 Grupo de Investigación en Sanidad Animal y Humana (GISAH), Carrera Ingeniería Agropecuaria, Departamento de Ciencias de la Vida y la Agricultura, Universidad de las Fuerzas Armadas ESPE, Sangolquí 171103, Ecuador; jwron@espe.edu.ec; 4 Department of Biomedical Sciences, Institute of Tropical Medicine, 2180 Antwerp, Belgium; ddbdb@telenet.be; 5 Facultad de Medicina Veterinaria y Zootecnia, Universidad Central del Ecuador, Quito 170521, Ecuador

**Keywords:** bovine, modelling, brucellosis, diagnosis, sensitivity, specificity, true prevalence, Bayes

## Abstract

In Ecuador, a national program for bovine brucellosis control has been in implementation since 2008. Given the costs, small- and medium-sized livestock holders are not completely committed to it. The objective of this study was to determine true prevalence (TP) of bovine brucellosis in small- and medium-sized herd populations, as well as the diagnostic sensitivity and specificity of the Rose Bengal (RB) test and the sero-agglutination test (SAT)-EDTA using a Bayesian approach. Between 2011 and 2016, 2733 cattle herds were visited, and 22,592 animal blood samples were taken in nineteen provinces on mainland Ecuador. Bayes-*p* and deviance information criterion (DIC) statistics were used to select models. Additionally, risk-factor analysis was used for herds according to their brucellosis test status. True prevalence (TP) in herds was estimated by pool testing. National seroprevalence of farms was 7.9% (95% CI: 6.79–9.03), and TP was 12.2% (95% CI: 7.8–17.9). Apparent prevalence (AP) in animals was 2.2% (95% CI: 1.82–2.67), and TP was 1.6% (95% CrI: 1.0–2.4). Similarly, the sensitivity of the RB was estimated at 64.6% (95% CrI: 42.6–85.3) and specificity at 98.9% (95% CrI: 98.6–99.0); for the SAT-EDTA test, sensitivity was 62.3% (95% CrI: 40.0–84.8) and 98.9% (95% CrI: 98.6–99.1) for specificity. Results of the two tests were highly correlated in infected and uninfected animals. Likewise, high spatial variation was observed, with the Coastal Region being the zone with the highest TP at 2.5%. (95% CrI: 1.3–3.8%) in individual animals and 28.2% (95% CI: 15.7–39.8) in herds. Risk factors include herd size, type of production (milk, beef, and mixed), abortions recorded, and vaccination. The results of this study serve to guide authorities to make decisions based on parallel testing at the beginning of a bovine brucellosis program for small livestock holders to increase sensitivity level of the screening tests in Ecuador.

## 1. Introduction

Bovine brucellosis is a highly contagious disease caused by *Brucella abortus*, less frequently by *Brucella melitensis*, and rarely by *Brucella suis*. The importance of this disease corresponds to its great economic impact on the livestock industry and serious risks related to human health [1].

Since 2008, Ecuador has a national program for the control of bovine brucellosis [2], which has contributed to reducing its prevalence. However, given its non-mandatory implementation and the high costs that owners must face for the diagnosis of the disease in animals, small- and medium-sized livestock holders (more than 80% of the producers in Ecuador) tend to not be involved in the program [3]. Likewise, Carbonero et al. [4] estimated the brucellosis seroprevalence in dairy and mixed cattle from semi-extensive systems in Ecuador as approximately 17.0% in individual animals and 45.1% at herd level. They used Rose Bengal (RB) and blocking ELISA tests for these analyses. Factors such as gender, dairy-herd type, closed facilities, non-ad libitum (restricted) feeding, age, average slope, and annual abortion rates were recognized as risk factors for the presence of seropositivity in cattle herds of Ecuador. In addition, Poulsen et al. [5] estimated an apparent prevalence of 5.5% and a true prevalence (TP) of 7.2% in animals based on a RB test. They determined higher prevalence of bovine brucellosis in commercial herds compared to smaller groups (of less than five animals). In conclusion, both studies stated that the overall prevalence is similar to other estimates obtained in cattle herds. Furthermore, in 1979, the National Animal Health Program (PNSA) of Ecuador carried out a serological survey to investigate the prevalence of bovine brucellosis, where 15,393 bovines were sampled. Using the rapid plate agglutination test, they obtained a seroprevalence of 6% (95% CI: 1.3–10) in Ecuador, ranging from 1.97% to 10.62% in cattle in the provinces of the Northern Highlands, from 4.12% to 10.62% in the Coastal Provinces, and from 1.3% to 2.6% in the provinces of the Southern Highlands [6]. The Ecuadorian Agency for Quality Assurance (Agrocalidad) together with a variety of representatives of organized milk producers and industrialists estimated that seroprevalence of brucellosis in animals from 1979 to 2008 is estimated at 3.30% [6]. Unpublished data obtained from distinct Ecuadorian universities show significant variability in cattle disease prevalence, ranging from 1–9.73% to 24–48% [5]. Thus, the estimation at both national and local levels might depend on the tests used, the testing strategy (e.g., serial or parallel testing procedure), and the sampling size [7]. For an effective surveillance of bovine brucellosis, reliable diagnostic tests must be used. Bacterial culture and isolation of the bacteria should be used to confirm (gold standard) the disease and determine which species or biovars of *Brucella* are causing it. However, the causative agent is a slow-growing microorganism. Therefore, the probability of recovering it is relatively low (around 20% in individuals with brucellosis) [8,9]. Additionally, the high costs, requirement of qualified personnel, and the need for high-security laboratories must be taken into consideration. [8,10,11]. In the absence of bacterial culture, several different serological tests offer a practical media for diagnosis. Furthermore, given that all tests have limitations, OIE recommends the use of multiple types of serological test for brucellosis diagnosis. Carrying out simultaneous serological tests to confirm the disease increases the sensitivity (Se) level and reduces the percentage of false negatives [12,13,14].

Due to all these drawbacks, screening populations in control programs requires the use of combined serological tests. Serological tests, such as RB and SAT, in the presence of EDTA might contribute to a rapid and practical diagnosis of brucellosis, given the possibility of exploring several kinds of antibodies [1,15,16,17]. The serum agglutination test (SAT) and the Rose Bengal test (RB) are commonly used for brucellosis screening in control and eradication programs. The Rose Bengal test is often used as a rapid and inexpensive screening test [15,16]. It is based on the agglutination of serum IgG antibodies with a preparation of whole cells stained with dead *Brucella*. RB has been used in the conventional and mass diagnosis of brucellosis. For confirmation of RB, SAT can be used in more sophisticated laboratories in addition to an enzyme immunoassay test (ELISA) [13,17]. SAT-EDTA particularly offers the possibility of detecting IgM for initial exposure [18].

Nevertheless, for massive programs of control, it is necessary to know the baseline of the disease; therefore, inexpensive, but reliable testing is necessary. A classic Rogan–Gladen equation is usually used to estimate the disease prevalence; unfortunately, it requires constant values and a prior knowledge of test sensibility (Se) and specificity (Sp), which may be unrealistic in practice. In Ecuador, very little is known about the test characteristics operating during brucellosis studies. In the two previous studies carried out in Ecuador [4,5], values for Se and Sp were reported given by the manufacturer or by literature estimates, respectively, with values of 98% and 99.9% for blocking ELISA, and 72.2% and 99.6% for the Rose Bengal test. These values were used to estimate the TP in Ecuador. On the other hand, Ron-Román et al. [18] reported sensitivities values for ELISA, RB, and SAT-EDTA tests in human samples of Ecuador with values around of 95.1%, 95.0%, and 60.8%, respectively. Specificity for these tests was >99.0%, but for SAT-EDTA, it was higher than 99.9%.

For this analysis, a Bayesian approach was used. Bayesian analysis involves calculating from a prior probability distribution and data likelihood, a posterior probability for the parameters of a given model [19,20]. Currently, the Bayesian approach is widely used for estimating the disease prevalence and the diagnostic characteristics of the tests (Se and Sp) jointly and in the absence of a gold standard test [21].

The present study aims to estimate the apparent and true prevalence of bovine brucellosis in small- and medium-sized livestock holders in Ecuador, as well as to determine the characteristics of two diagnostic tests (Se and Sp) for RB and SAT-EDTA. Another aim was to identify the risk factors that predispose the presence of this disease in Ecuadorian cattle farms in order to formulate some recommendations for the national control program of bovine brucellosis [22]. All these results of this study will improve Ecuador’s public health and livestock economy by encouraging the consideration and implementation of effective brucellosis control strategies, based on national data, since elaborating programs cannot be created based on the extrapolation of international data [23].

## 2. Materials and Methods

### 2.1. Description of the Study

This cross-sectional study was part of the brucellosis, tuberculosis, and cattle ticks national survey that started in 2012 and ended in 2015 on mainland Ecuador [24]. The study population consisted of agricultural productive units (farms) that raise cattle, with an emphasis on small and medium herds. The bovines were selected by random sampling in small, medium, and large farms belonging to 19 of the 24 provinces in Ecuador. The selection of the number of animals to sample by province was carried out by taking into account the number of animals that the province contributes to the national total of animals (weighting). Both female bovines (with or without RB51 vaccine) and male bovines older than 6 months were sampled. Female bovines vaccinated with Strain 19 that were older than 18 months were also sampled [25].

The animals were sampled without distinction of the breed, choosing a proportion according to the number of animals present on each farm: 4–6 animals (75%), 7–15 animals (50%), 16–30 animals (33%), 31–80 animals (29%), 81–160 animals (25%), and more than 160 animals (40 animals) [26]. Blood samples were taken from 22,592 animals, belonging to 2733 livestock farms, which were classified as large (more than 70 cattle), medium (21 to 70 cattle), and small (1 to 20 cattle).

Cattle farms corresponding to 19 provinces, which were classified into regions: the Coastal Region (Esmeraldas, Guayas, Los Ríos, Manabí, Santa Elena, and Sto. Domingo), the Northern Highlands (Bolívar, Carchi, Chimborazo, Cotopaxi, Imbabura, Pichincha, and Tungurahua), the Southern Highlands (Azuay, El Oro, Loja, and Zamora Chinchipe), and the Amazon Region (Napo and Pastaza). This classification takes into account similar production systems and the provincial population size of the herds. The sampled provinces are shown in Table 1.

To evaluate the risk factors, an epidemiological survey was applied on each farm, which consisted of a personal interview with the people in charge. This questionnaire included aspects related to the identification and location of the farm, general data (number of animals and production type), and sanitary aspects (reproduction system, abortion management, veterinary control, clinical manifestations of the disease, diagnosis, and vaccination).

### 2.2. Diagnostic Assays

Blood samples were collected from each animal by coccygeal venipuncture. The samples were labeled and transported to the laboratory on ice (4–8 °C) after clotting. The blood serum obtained by centrifugation (250 rpm) was processed and analyzed in the immunodiagnostic laboratory at the International Zoonoses Institute (CIZ) at the Central University of Ecuador. The screening tests used to determine the presence of antibodies against *Brucella* spp. were RB and the serum agglutination test in the presence of EDTA (SAT-EDTA) with a cut-off point above 30 IU (International Units) (25% of agglutination for the dilution 1/25), according to OIE and Sciensano protocols. Unidentified, hemolyzed, icteric, lipemic, and contaminated samples were discarded [27] and labelled as “Not Reported” (NR); samples of animals that had an NR result in both RB and SAT-EDTA were discarded. Finally, a cured database was obtained with the results of 22,126 animals (97.9% sampled) belonging to 2733 farms (100% sampled).

### 2.3. Statistical Analysis

Apparent prevalence at animal level: this was calculated for the two tests using parallel interpretation; 95% interval confidence for the proportion estimation was obtained.

Parallel interpretation: An animal was considered positive when at least one of the tests gave a positive result. That is, it was sufficient that one of the tests was positive in order to declare that animal as sick, regardless of the information provided by the other tests [28,29]. Parallel tests increased Se and negative predictive value (NPV) and decreased Sp and positive predictive value (PPV) [30,31].

True prevalence at animal level and test characteristics: Two Bayesian frameworks, probabilistic constraints [32] and co-variances between tests [33], were created to estimate this prevalence. Functions truePrevMulti and truePrevMulti2 from the Prevalence Package [34] were used for this propose under R environment 3.5.2 [35]. The two models were used to calculate the true prevalence and the test characteristics. Likewise, evaluation of test co-variances for the truly infected and uninfected animals under Model 2 Markov Chain Monte Carlo (MCMC) was done by using the freeware program WinBUGS 1.4.3 [36] (Appendix A-Table A1).

For the first model, which used a conditional probability scheme, a total of 7 parameters (*θ_i_*) were required [32,34]. Prior information on the prevalence of brucellosis in Ecuador [3,6] and the characteristics of RB, sensitivity and specificity [37], was obtained from several previous studies. The parameters used in this model were:

$$\theta_{1}=TP=P\left(D^{+}\right);\;\theta_{2}=Se_{RB}=P(T_{1}^{+}|\;D^{+});\;\theta_{3}=Sp_{RB}=P(T_{1}^{-}|\;D^{-});\theta_{4}=P(T_{2}^{+}|\;D^{+}\cap T_{1}^{+});\;\theta_{5}=P(T_{2}^{-}|D^{-}\cap T_{1}^{-};\;\theta_{6}=P(T_{2}^{+}|\;D^{+}\cap T_{1}^{-})\;\mathrm{and}\;\theta_{7}=P(T_{2}^{-}|\;D^{-}\cap T_{1}^{+})$$

where *P* *=* probability; $D^{+}\;$= animals with brucellosis; $D^{-}\;$= healthy animals; $T_{1\;}^{+}\;$= positive animals to the RB; $T_{1}^{-}\;$= negative animals to the RB; $T_{2}^{+}\;$= positive animals to the SAT-EDTA; $T_{2}^{-}\;$= negative animals to the SAT-EDTA. For some parameters ($\theta_{4},\;\theta_{5},\theta_{6},\;\theta_{7}$), it was not possible to obtain objective prior information. Therefore, it was necessary to consider the previous information on these parameters as “non-informative” (Table 2).

For the second model, which used a covariance scheme between tests [34], seven parameters were required. The model construction strategy consisted of incorporating prior information on the prevalence of brucellosis in Ecuador [3,6] and on the characteristics of the RB, sensitivity and specificity [37], and SAT-EDTA, sensitivity and specificity [38], which were obtained from several previous studies (Table 2). The parameters used in this model were:

$$\theta_{1}=TP;\;\theta_{2}=Se_{RB};\;\theta_{3}=Sp_{RB};\;\theta_{4}=Se_{SAT-EDTA}=P(T_{2}^{+}|D^{+});\;\theta_{5}=Sp_{SAT-EDTA}=P(T_{2}^{-}|\;D^{-});\;\;Cov\;(T_{1},T_{2}|\;D^{+})=a=\theta_{2}\theta_{4}-\left(\theta_{2}\left(\theta_{2}\theta_{4}+\theta_{5}-\theta_{2}\theta_{5}\right)\right)\;\mathrm{and}\;Cov\;(T_{1},\;T_{2}|\;D^{-})=b=\left(1-\theta_{3}\right)-\;\left((1-\theta_{3})\left(1-\theta_{7}+\theta_{3}\theta_{7}-\theta_{3}\theta_{6}\right)\right).$$

Due to covariances, it was not possible to formulate objective prior information; therefore, it was necessary to regard the previous information on these parameters as “non-informative” (Table 2). Additionally, for each region in Ecuador, different models with varied prior information were run (Appendix A-Table A1 and Table A2). The two models were run using three MCMC chains, a burn-in period of 1000 iterations, and another 15,000 iterations. The convergence of each model was evaluated by using density plot, trace plot, Brooks–Gelman–Rubin (BGR) plot, and autocorrelation plot. Model selection was carried out according to the criteria previously described in the literature [28,32]. The measure of the compatibility of the model was verified by the Bayes-*p* statistic, which should be as close as possible to 0.50. Substantially different values indicated a lack of convergence of the model [34]. The deviance information criterion (DIC) guaranteed the most parsimonious model, and models with a smaller DIC were chosen as an indication of the probable parameter–space solutions [18].

Agreement between diagnostic tests: Both the Kappa coefficient and the positive and negative agreement between the tests were obtained. The Kappa coefficient was calculated by using cohen.kappa function from the psych Package [39]. The level of agreement was expressed in terms of indices of positive and negative agreement [40]. Confidence intervals were calculated as described by Uebersax in 2018 [41].

True prevalence at farm level: A farm was considered positive when at least one animal tested positive, either to RB $\left(T_{1}^{+}|\;T_{2}^{-}\right)$, SAT-EDTA $\left(T_{1}^{-}|\;T_{2}^{+}\right)$ or to both tests $\left(T_{1}^{+},T_{2}^{+}\right)$. For the true prevalence at farm level (π), the results of the Bayesian analysis were used. The average of the total farm samples was pool size (*k*) as described by [19], where *P* is the brucellosis seroprevalence at herd level.

π=1−1−P1k

Risk factors for test seropositivity on farms: Factors investigated were herd size, type of production (milk, beef and mixed), permanent veterinary control, reproduction system (artificial insemination, natural breeding, mixed), abortions recorded, and brucellosis vaccination. Factor influence was determined by using multiple logistic regression. The final adjusted model was obtained using glm function and stepAIC function from the MASS package [42] in R environment.

## 3. Results

### 3.1. Apparent Prevalence at Animal Level

**Figure 1:**
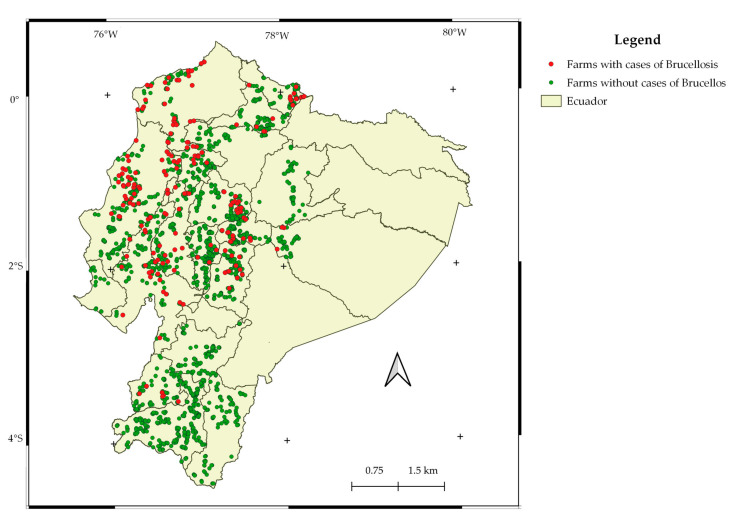
Farms with and without cases of brucellosis.

Apparent prevalence, based on SAT-EDTA and RB results, in addition to parallel testing, yielded similar results of approximately 2% of animals. Sampled farms with results from tests and a layer with the provincial administrative country division are presented in Figure 1. Table 3 presents seroprevalence per study zone.

### 3.2. Agreement between Tests

To measure the agreement between the RB and SAT-EDTA, a kappa index of 0.93 (95% CI: 0.92 to 0.95) was obtained. The index of positive agreement was 0.936 (95% CI: 0.910–0.953), and the index of negative agreement was 0.999 (95% CI: 0.998–0.999).

### 3.3. True Prevalence at Animal Level

The results of the cross classification of the two tests on the 22,126 animals are shown in Table 4. Of the total of the samples analyzed, 440 were positive to SAT-EDTA, of which 419 were also positive for RB.

Table 5 presents the Bayesian estimations for parameters obtained, on a national level, by the two models proposed and their 95% credibility intervals (CrI) with respect to the true prevalence and test characteristics. Appendix A-Table A3 presents the results by regions.

True prevalence was estimated to be around 1.6% (95% CrI: 1.0–2.3). Furthermore, there was evidence of spatial variability due to disease prevalence. The Coastal Region had the highest true prevalence of 2.5% (95% CrI: 1.3–3.8), whilst the Southern Highlands had the lowest prevalence at the animal level of 0.1% (95% CrI: 0.0–0.3).

**Figure A2:**
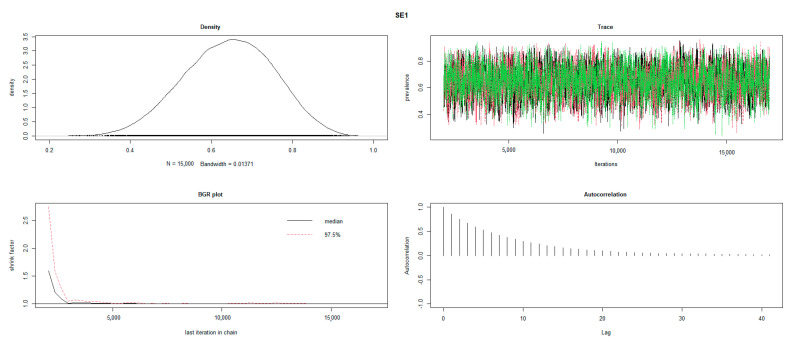
Density, trace, BGR plot, and autocorrelation for the sensibility of the Rose Bengal test in Ecuador.

**Figure A3:**
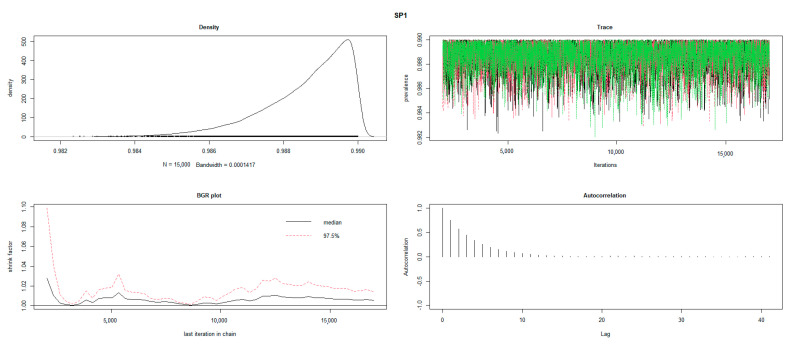
Density, trace, BGR plot, and autocorrelation for the specificity of the Rose Bengal test in Ecuador.

**Figure A4:**
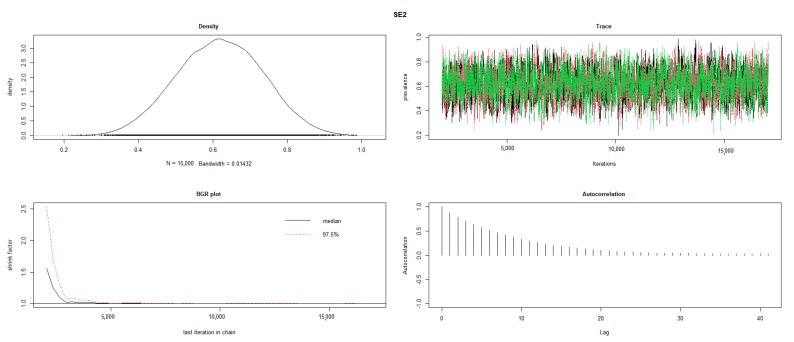
Density, trace, BGR plot, and autocorrelation for the sensibility of the SAT-EDTA test in Ecuador.

**Figure A5:**
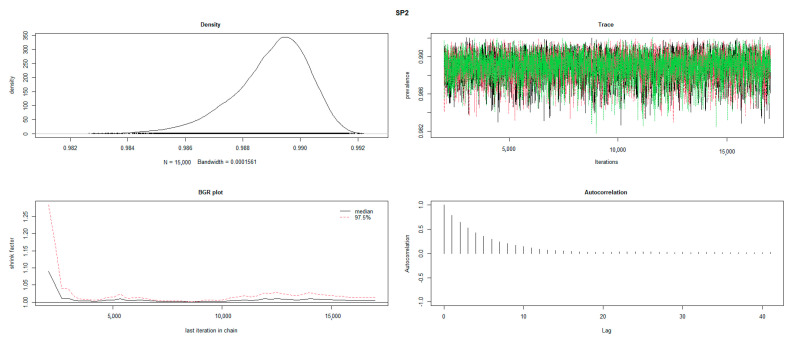
Density, trace, BGR plot, and autocorrelation for the specificity of the SAT-EDTA test in Ecuador.

In general, both models gave similar results. However, convergence statistics were favorable to Model 1 (with probabilistic constraints), while BGR values were close to 1. The trace plot of the chains was mixed correctly, and density plots retained a unimodal and Gaussian bell shape (Appendix A-Figure A1, Figure A2, Figure A3, Figure A4 and Figure A5). Values obtained for Bayes-*p* and DIC showed that Model 1 was more parsimonious, since all the Bayes-*p* values were close to 0.5 (the values ranged from 0.52 to 0.66) and presented smaller DIC values in comparison to Model 2 (Table 5 and Appendix A-Table A2 and Table A3).

### 3.4. Test Characteristics of RB and SAT-EDTA

The sensitivity and specificity estimations for RB and SAT-EDTA were similar in the two models throughout all regions (see Table 5 and Appendix A-Table A3). RB sensitivity, however, varied among the regions. On a national level, it was 64.5% (95% CrI: 43.1–84.9), but it was higher in the Amazon Region with 81.8% (95% CrI: 64.0–94.5), followed by the Highland Regions with 73.1% (95% CrI: 53.2–89.0), and lowest in the Coastal Region with 72.4% (95% CrI: 51.4–89.5). Estimations of SAT-EDTA sensitivities in the regions were slightly lower than the RB. On a national level, it was 62.2% but with wider 95% CrI as 40.6–84.2. Similarly, among the different regions, it reached the highest value in the Amazon Region where it was 81% (95% CrI: 64.2–93.8), and the lowest sensitivity occurred in the Southern Highlands, i.e., 59.4% (95% CrI: 33.9–85.5). Overall, the sensitivity estimations show informative and unimodal posterior distributions. Conversely, specificity estimations were relatively high (over 98%) for both tests, being 99% in the majority of cases.

The sensitivity and specificity estimations for RB and SAT-EDTA in Ecuador in parallel testing were, respectively, 67.63% (95% CrI: 45.14–89.53) and 98.81% (95% CrI: 98.51–98.97).

### 3.5. Relationship between Test Results and the Status of the Disease

The values of the covariants a and b are similar in the two models, as observed in Table 5 and Appendix A-Table A3. In the case of covariant a (covariance between tests in infected individuals), it was 0.18 in the whole country, varying from 0.15 in the Coastal Region to 0.03 in the Southern Highlands, and 0.06 in the Amazon Region. This positive covariance in the infected individuals has a probabilistic interpretation that can be calculated $P(T_{2}^{+}|T_{1}^{+},D^{+})$ by using these covariances. These probabilities were 0.90 for all of Ecuador, 0.93 for the Coastal Region, 0.88 for the Northern Highlands, 0.75 in the Southern Highlands, and 0.79 in the Amazon Region. In the last two cases, the tests were more independent, and conversely, as in the case of the whole country, a positive result tends to be positive in the second test. Similarly, covariant b (covariance between tests in uninfected individuals) showed relatively lower values going from 0.002 to 0.01, which in terms of conditional probabilities ($P(T_{2}^{-}|T_{1}^{-},D^{-})$ represented values near 0.98 or higher. This reflects the high correlation between negative results.

### 3.6. Prevalence at Animal Level on Farms with and without Vaccination

Out of 22,126 animals, 4589 belong to farms that were vaccinated previously against brucellosis. In Ecuador, two types of vaccines, nationally produced Strain 19 and imported biological RB51, are used in cattle for the prevention of brucellosis. Sixty-six percent of farms that vaccinated used Strain 19, 18.5% used RB51, and 15.5% do not know which vaccine they used. The values of true prevalence and diagnostic test characteristics of RB and SAT-EDTA of vaccinated and unvaccinated animals are shown in Table 5.

### 3.7. Apparent and True Prevalence at Herd Level

Herd-apparent and true prevalence estimations are shown in Table 3 for both the entire country, as well as for the different regions. Brucellosis-true prevalence for farms varied from almost 0.1% in the Southern Highlands to 28% in the Coastal Region. On a national level, apparent prevalence for farms was 7.9% (95% CI: 7–9), and the true prevalence was 12.2% (95% CI: 7.8–17.9). The average pool sizes (*k*) for those true prevalence estimates were *k* = 8 nationally, *k* = 13 for the Coastal Region, *k* = 6 for the Northern Highlands, *k* = 7 for the Southern Highlands, and *k* = 9 for the Ecuadorian Amazon Region.

### 3.8. Risk Factors for Farm Seropositivity

Six variables were significant in the initial selection of variables as potential risk factors associated with brucellosis seropositivity on farms. These included: farm size, type of production, veterinary control, reproduction system, abortions recorded, and vaccination on the farm (Table 6).

After stepwise process selection, the chosen model was the one with less AIC (1159.19) that included four risk factors: farm size, type of production, abortions recorded, and vaccination (Table 6). Medium and small farms were less affected than large farms (*p*-value < 0.001): OR = 0.457 (95% CI: 0.310–0.671) and 0.17 (95% CI: 0.107–0.271). Mixed (milk and beef) and dairy farms were more affected than beef cattle farms with OR = 1.78 (95% CI: 0.939–3.375). Farms that had carried out brucellosis vaccination had a higher risk (OR = 1.895; 95% CI: 1.375–2.612) than farms without vaccination. Finally, farms with abortions recorded had a higher risk (OR = 3.13; 95% CI: 2.172–4.509) than farms without the presence of abortions.

## 4. Discussion

Brucellosis is one of the most common zoonoses in the world. It has proven to be a great problem for public health in developing countries, affecting both humans and animals. Furthermore, it has generated great economic losses in the livestock industry. Although the disease produces reproductive problems, many animals remain asymptomatic, so several diagnostic tests are needed to determine the presence of the disease [1,8,18,43,44].

In this study, a Bayesian approach was used to estimate cattle brucellosis prevalence and the test characteristics (sensitivity and specificity) of two serological tests [18] by using two approaches: one based on conditional probabilities [32] and the other based on covariances between tests [28]. Bayesian approach allowed the incorporation of external information based on expert opinions and the bibliographic compilation of previous studies [32,45]. In practice, accessing prior knowledge made it possible to know estimations of the model parameters. Therefore, prior information should be sought and incorporated in terms of prior distributions [46,47]. Additionally, the large sample size (22,126 animals) made a corrective strategy, which allowed the data “to speak for itself” [48]. In this sense, Model 1 required fewer parameters than Model 2, which would have required prior knowledge of sensitivities and specificities for both tests. Thus, Model 1 obtained lower DIC values in all the cases and also obtained better predictive posterior probabilities (approximately 0.50) in contrast to Model 2, in which Bayes-*p* values were far from 0.50.

In addition, this research focused on determining the presence of brucellosis on small and medium cattle farms, since they encompass 86.7% of farms nationwide. Apparent prevalence was estimated in 2.2% with the TP was 1.6% (95% CrI: 1.0–2.4) at the animal level and 12.2% at the farm level. The apparent prevalence at the farm level as 7.9% (95% CI: 7.0–9.0) agreed with results obtained in a study where 2054 animals were sampled. Indeed, 5.19% (101/2054) were positive for RB and 6.49% (126/2054) positive for SAT-EDTA (Ron-Román J., 2014, unpublished data). By comparing the true prevalence obtained (1.6%) with that estimated in 1979 [6] and the one from 2008 (3%) [3], it can be concluded that the National Program for the Control of Brucellosis implemented by Agrocalidad decreased the number of cattle infected with bovine brucellosis. However, continued presence of the disease may be due to the fact that the certification offered is not compulsory. Certification also tends to be obtained more frequently by large farms, omitting numerous small and medium farms. Other study mentions that although small producers know about the dangers of the disease, they will not separate the animals that have been aborted from the rest of the cattle, since they do not have the necessary physical facilities to isolate sick or suspicious animals [49]. Additionally, the extra payment offered by participating in this program (US$0.01 per liter) [25] might not be significant enough to encourage animal brucellosis testing by small farmers.

Carbonero et al. [4] in a random cross-sectional study took 2666 samples from 386 dairy and dual-purpose cattle farms in eight provinces of Ecuador (Azuay, Chimborazo, Cotopaxi, Manabí, Pichincha, Santo Domingo, Tungurahua, and Zamora Chinchipe) analyzed by using RB test and a blocking ELISA test determined that the true prevalence in Ecuador is 17% (95% CI: 15.6–18.4%) at the animal level and 45.1% at the farm level. This study included regional coverage, while in previous years, there were only local studies available that had been carried out as part of university undergraduate theses research. The results published by Carbonero [4] differ from those obtained in this study, since the authors took sensitivity and specificity values provided by the manufacturer. It is currently known that these values vary due to external factors, as well as according to the field in which they are applied [32,50,51,52]. This variation is mainly due to the differences between reference populations, sampling strategies that have been used for the validation procedure, technical variation of the tests (distinct laboratories), competence of the laboratory, choice of gold standard and cut-off value for interpretation and management of results, and the sanitary conditions of the populations with respect to other conditions [53].

This study found that the areas with the highest true prevalence of brucellosis in Ecuador are the Coastal Region and Northern Highlands with prevalence levels around of 2.5% and 1.0%, respectively, these results maintain the classification given by Torres in 2008 [3], in which the Coastal Region and the Northern Highlands are considered regions of high prevalence. However, in the Northern Highlands, brucellosis prevalence has decreased as the main dairy farms are here and receive a bonus of US$0.01 per liter of raw milk for herds certified as free of brucellosis in order to encourage and promote animal health in the national dairy herd [54].

In contrast, the Coastal Region had an increase in seroprevalence because the exploitation of beef and mixed cattle predominates here [55]. Since dairy is not the main activity, farmers are more reluctant than dairy producers to apply control measures because they do not benefit from of the brucellosis-free herd bonus [56]. Previous study reported that beef-producing breeds are 22 times more likely to contract the disease than breeds dedicated to milk production, since these breeds are usually mobilized without any control [57]. Furthermore, the mixture of breeds incentivizes the mobilization of animals from different regions. For example, dairy bulls are transported to tropical zones to improve milk production. Moreover, extensive farming and low technological development might have also caused increased brucellosis intensity in that region [58].

The Southern Highlands and Amazon Region presented true prevalence values of 0.1% and 0.8%, respectively, considered as low-prevalence regions. The Southern Highlands presented a lower prevalence, probably due to the number of small farms and limited commercial integration with the epidemiological regions of high prevalence, since the commercialization of animals is carried out internally in the region [3,6]. In the Amazon Region, the prevalence is relatively low; however, herds with mixed breeds are prominent in this region and, as the risk analysis confirmed, more likely to be seropositive [57,59].

In comparing the results of the Northern and Southern Highlands, it can be determined that the true prevalence is higher in the Northern Highlands due to the high mobility of allegedly infected animals. This can be contributed to the fact that it is a borderland with commercial exchange between Ecuador and Colombia [60], a country that records a seroprevalence of bovine brucellosis of 2.4% to 5% [61].

The risk variables associated with the presence of bovine brucellosis in 2733 herds sampled in Ecuador were farm size, type of production, abortions recorded, and vaccination on the farm. The seroprevalence increased significantly with herd size, since large herds have a higher risk of seropositivity in comparison to the small and medium ones. Certain characteristics of large herds, such as difficulty of individualized handling of animals and deficient sanitary control, increases the exposure potential, especially after incidents of abortions in which there is contact and common feeding at watering points, which promotes the transmission of *Brucella* organisms [62,63,64].

A positive association was found between the presence of previous abortions with brucellosis seropositivity. This could be explained by the fact that abortion is a typical outcome in brucellosis-infected animals [63]. Mixed herds (dairy–beef) had a higher risk of becoming infected than farms with dairy or beef-only herds (OR = 1.78, using beef cattle as the reference). This is due to the fact that herds of mixed breeds are associated with a higher number of seropositive cattle in a herd and, because of the mixture, increase animal mobilization [65].

It can be observed that the true prevalence in vaccinated animals (2.6% with 95% CrI: 1.0–4.5%) is almost double in comparison with unvaccinated animals (1.4% with 95% CrI: 0.7–2.2%), indicating that positives are associated with vaccination or the presence of true positive responses (due to infection) in a context of an unappropriated vaccination control program on farms. Vaccinating animals with inappropriate doses, at incorrect ages and unrecommended administration procedures, also represents a risk for abortion or premature birth, especially when using Strain 19 [66]. The specificity values of the tests that include unvaccinated animals are higher than in populations where animals have received vaccination as well as in the general sampling (with and without vaccination). This is due to the fact that the number of false positives decreases by doing the more specific tests [51]. However, the specificity does not reach 100%, since false positives may also be due to the probable presence of cross reactions such as *Yersinia enterocolitica* O: 9, *Escherichia coli* O: 157, *Salmonella* group N (O: 30), *Vibrio cholerae* O1 infections, *Scherichia hermani*, or *Stenotrophomonas maltophilia* [67,68]. Additionally, sensibility and specificity values of the two serological tests had similar values to the ones previously reported when this division was not carried out.

However, according to the results, 65% of vaccinated animals have used Strain 19. This vaccine has a smooth phenotype, making it difficult to distinguish between vaccinated and naturally infected animals through the use of common serological diagnostic tests, especially if the vaccination is not exclusively reserved for the young animals [69,70]. Additionally, this vaccine is an alive vaccine that can cause a reaction as described in goats by [71] and humans [72]. This can also be associated with a poor vaccination system, especially in rural areas [73]. According to another study, Strain 19 is excreted intermittently in milk throughout a reproductive cycle in of dairy cows [74]. In addition, *Brucella* spp. DNA was detected by multiplex PCR in 37/192 fresh cheese, where 30 samples were classified as Strain 19 [66], illustrating a real risk to public health.

Although brucellosis prevalence has decreased at both the national and regional levels, it is important to update the information on the epidemiological status of each region in order to identify aspects of the brucellosis control program that should be improved or revamped. Furthermore, this should be accompanied by health education for the producers and veterinarians regarding biosecurity and efficient vaccination systems, since limited knowledge makes it impossible to achieve the elimination of the disease in cattle and humans [56,59,75].

Although there are several studies to determine the cattle-brucellosis prevalence in Ecuador, there are no studies on the test characteristics (sensitivity and specificity) of the RB and SAT-EDTA tests. Knowing the true values of Se and Sp will help provide better diagnosis of the disease by reducing the incorrect classification of infected and uninfected cattle. This will also prevent unnecessary economic losses when animals are wrongly classified by tests [76].

The incorporation of SAT-EDTA would increase the quality of the national control program, since 11% (θ_5) more animals with acute infection may be diagnosed. In this sense, parallel testing at the beginning of a control program is necessary because it increases the sensitivity level and helps eliminate true positives more quickly. The combination of SAT and iELISA (parallel testing) in risk and restricted herds is being used widely in brucellosis program elsewhere, as it increases the number of diagnosed cases and allows for follow-up [77,78]. These results suggest that Ecuador should change tests in the national brucellosis control program for tests capable of detecting different stages of the disease (IgG and IgM).

According to the results obtained, it can be assumed that the RB and the SAT-EDTA tests are conditionally dependent on the true animal-health status. This is due to the fact that both tests may have a similar biological basis and SAT-EDTA might also distinguish IgG immunoglobulins in addition to IgM. Therefore, the assumption of conditional independence is unsustainable due to the similarity of antibodies detected by the two tests [32,79,80]. In this sense, the results should be highly correlated, and the sensibility of the combination not increase as much as if they were independent. The concordance indices of the results of the two tests indicate a high level of agreement. The kappa statistic was 0.93 (95% CI: 0.92–0.95), a value considered almost perfect as described by Landis and Koch [81]. In the assessment of the test agreements, the indices of positive and negative agreement results were 0.936 (95% CI: 0.920–0.953) and 0.999 (95% CI: 0.998–0.999), respectively, which reinforces the aforementioned. In the case of positive results, only 5% of cases do not agree; in the case of negative results, less than 1% do not agree.

The diagnostic test characteristics at the national level of RB (Se = 64.5% with 95% CrI: 43.1–84.9; Sp = 98.9% with 95% CrI: 98.6–99.0%) agree with the findings previous studies [38,45,82]. The sensitivity of SAT-EDTA (62.2%; 95% CrI: 40.6–84.2) and specificity 98.9% (95% CrI: 98.6–99.1%) agree also with the results of previous studies [38,83,84,85]. The results of this investigation varied slightly according to each region where the study was carried out (Coastal Region, Northern Highlands, Southern Highlands, or Amazon Region) due to host factors, infectious dose, and distribution of biological factors related to the infection (stage and severity of the disease) [38,86,87]. Parallel testing strategy presented a Se of 67.63% (95% CrI: 45.14–89.53) and Sp of 98.81% (95% CrI: 98.51–98.97), which may be useful for massive testing to reduce brucellosis prevalence at the initial stages of the control program where high rates of prevalence are reported. Furthermore, this strategy can be implemented at a relatively low cost, which can be affordable for small and medium cattle farms.

## 5. Conclusions

Based on two serological tests of relatively quick implementation and a large-enough sample size, cattle brucellosis true prevalence was estimated in Ecuador to be around 1.6% at the animal level and 12% at the farm level. Geographic spatial variations were identified around the country, the Coastal Region presenting higher prevalence (2.5%). Risk-factor analysis found that mixed production and the abortions recorded on the farms increased the risk of cattle brucellosis presence on farms. The true prevalence in vaccinated animals (2.6% with 95% CrI: 1.0–4.5%) is almost double in comparison with unvaccinated animals (1.4% with 95% CrI: 0.7–2.2%). The Strain 19 vaccine was used in sixty five percent (65%) of vaccinated animals.

## Figures and Tables

**Table 1:** Farms samples.

Parameter	Sample			Positive **		
	N°	%	Mean *	N°	%	Mean *
COASTAL REGION (Animals)	9355	42.28	13.08	310	65.13	2.33
COASTAL REGION (APUs)	715	26.16	-	133	61.29	-
Large Farms	165	6.03	-	61	28.11	-
Medium Farms	325	11.89	-	57	26.27	-
Small Farms	212	7.76	-	12	5.53	-
Not reported	13	0.48	-	3	1.38	-
NORTHERN HIGHLANDS (Animals)	6880	31.09	5.6	139	29.2	1.96
NORTHERN HIGHLANDS (APUs)	1229	44.97	-	71	32.72	-
Large Farms	52	1.91	-	11	5.07	-
Medium Farms	175	6.4	-	23	10.6	-
Small Farms	985	36.04	-	36	16.59	-
Not reported	17	0.62	-	1	0.46	-
SOUTHERN HIGHLANDS (Animals)	4767	21.54	7.13	11	2.31	1.38
SOUTHERN HIGHLANDS (APUs)	669	24.48	-	8	3.69	-
Large Farms	30	1.1	-	0	0	-
Medium Farms	175	6.4	-	3	1.38	-
Small Farms	410	1.5	-	3	1.38	-
Not reported	40	1.47	-	1	0.46	-
AMAZON REGION (Animals)	1124	5.08	9.37	16	3.36	3.2
AMAZON REGION (APUs)	120	4.39	-	5	2.3	-
Large Farms	3	0.11	-	1	0.46	-
Medium Farms	59	2.16	-	1	0.46	-
Small Farms	55	2.01	-	3	1.38	-
Not reported	3	0.11	-	0	0	-
Total General (Animals)	22,126	100	8.1	476	100	2.19
Total General (APUs)	2733	100	-	217	100	-

Legend: AUP, animal unit production; Mean * = mean number of animals per APU (sampled or positive); ** an animal is considered positive when at least one test result was positive (parallel interpretation).

**Table 2:** Prior information used for sensitivity and specificity of RB and SAT and prevalence in Ecuador used in Models 1 and 2.

MODEL 1			MODEL 2			REFERENCES
Parameter	Value	Distribution	Parameter	Value	Distribution	
theta [1]	3%	Beta = (16, 486)	TP	3%	Beta = (16, 486)	[3]
theta [2]	72%	Beta = (16, 6)	SE [1]	72%	Beta = (16, 6)	[37]
theta [3]	71–99%	Uniform = (0.71, 0.99)	SP [1]	71–99%	Uniform = (0.71, 0.99)	[37]
theta [4]	NI	Uniform = (0.00, 1.00)	SE [2]	50–99%	Uniform = (0.50, 0.99)	[2,38]
theta [5]	NI	Uniform = (0.00, 1.00)	SP [2]	90–100%	Uniform = (0.90, 1.00)	[38]
theta [6]	NI	Uniform = (0.00, 1.00)	a [1]	NI	Uniform = (0.00, 0.25)	-
theta [7]	NI	Uniform = (0.00, 1.00)	b [1]	NI	Uniform = (−0.25, 0.25)	-

Legend: NI = non-informative.

**Table 3:** Estimations of the apparent prevalence of brucellosis in animals and at the farm level (%) in Ecuador and in the Coastal Region, North Highlands, South Highlands, and Amazonia.

Region	Apparent Prevalence (95% CI) Animal Level		Apparent Prevalence (95% CI) Farm Level *	True Prevalence (95% CI) Farm Level *
Ecuador	RB	2.10 (1.72–2.56)	7.9 (7.0–9.0)	12.2 (7.8–17.9)
	SAT-EDTA	2.03 (1.66–2.49)		
	Parallel interpretation	2.20 (1.81–2.67)		
Coastal Region	RB	3.29 (2.56–4.22)	18.6 (15.9–21.7)	28.2 (15.7–39.8)
	SAT-EDTA	3.15 (2.44–4.06)		
	Parallel interpretation	3.43 (2.69–4.36)		
Northern Highlands	RB	1.97 (1.40–2.78)	5.8 (4.6–7.3)	5.5 (2.2–9.2)
	SAT-EDTA	1.91 (1.33–2.74)		
	Parallel interpretation	2.03 (1.45–2.84)		
Southern Highlands	RB	0.23 (0.11–0.49)	1.2 (0.6–2.4)	0.7 (0.6–2.1)
	SAT-EDTA	0.21 (0.09–0.47)		
	Parallel interpretation	0.23 (0.11–0.49)		
Amazon Region	RB	1.17 (0.30–4.57)	4.2 (1.5–9.9)	7.2 (0.9–15.6)
	SAT-EDTA	1.44 (0.45–4.68)		
	Parallel interpretation	1.44 (0.45–4.68)		

Legend: *A farm is considered positive when it has one or more animals that had positive result on the RB or SAT-EDTA test (parallel interpretation).

**Table 4:** Cross-classification of the RB and SAT-EDTA test results for cattle brucellosis in Ecuador based on RB and SAT-EDTA.

	$T_{2}^{+}$	$T_{2}^{-}$	Total
$T_{1}^{+}$	419	36	455
$T_{1}^{-}$	21	21,650	21,671
Total	440	21,686	22,126

**Table 5:** Posterior means and 95% credibility intervals (CrI) of the true prevalence and test parameters obtained from two different models in Ecuador.

Parameter	With and without Vaccination		With Vaccination	Without Vaccination
	Model 1	Model 2		
	Mean (95% CrI)	Mean (95% CrI)	Mean (95% CrI)	Mean (95% CrI)
TP	1.6% (1.0–2.3%)	1.5% (1.0–2.0%)	2.6% (1.0–4.5%)	1.4% (0.7–2.2%)
$Se_{T1}$	64.5% (43.1–84.9%)	65.2% (51.9–82.6%)	79.4% (60.4–92.7%)	77.0% (57.0–91.9%)
$Sp_{T1}$	98.9% (98.6–99.0%)	98.9% (98.6–99.0%)	98.1% (96.7–99.7%)	99.6% (99.0–99.9%)
$Se_{T2}$	62.2% (40.6–84.2%)	63.0% (50.8–81.9%)	80.8% (60.0–95.7%)	77.8% (56.7–94.5%)
$Sp_{T2}$	98.9% (98.6–99.1%)	98.9% (98.6–99.1%)	98.1% (96.6–99.7%)	99.5% (98.9–99.9%)
cov_a	0.176 (0.074–0.234)	0.179 (0.090–0.232)	0.108 (0.006–0.207)	0.123 (0.014–0.220)
cov_b	0.011 (0.010–0.014)	0.010 (0.009–0.013)	0.018 (0.003–0.032)	0.004 (0.000–0.010)
BGR	1.00	1.01	1.01	1.00
Bayes-*p*	0.55	1.00	0.54	0.52
DIC	21.66	39.63	18.18	19.84

Legend: $Se_{T1}$ = sensibility of Rose Bengal test; $Sp_{T1}$ = specificity of Rose Bengal test; $Se_{T2}$ = sensibility of SAT-EDTA test; $Sp_{T2}$ = specificity of SAT-EDTA test; cov_a = relationship: RB and SAT-EDTA/disease; cov_b = relationship: RB and SAT-EDTA/absence of disease; BGR = multivariate Brooks–Gelman–Rubin (BGR) statistic; Bayes-*p* = Bayesian *p*-values; DIC = deviance information criterion; CrI = credibility interval.

**Table 6:** Risk factors associated with cattle brucellosis seroprevalence in farms after multivariate analysis in Ecuador.

Risk Factor		N° Farms	Positive Farms	Seropos	Initial Model		Final Model	
					OR (95% CI)	*p*-Value	OR (95% CI)	*p*-Value
Farm size	Large	264	74	28.0%	Reference			
	Medium	734	84	11.4%	0.462 (0.313–0.681)	<0.001	0.457 (0.310–0.671)	<0.001
	Small	1662	54	3.2%	0.176 (0.110–0.281)	<0.001	0.170 (0.107–0.271)	<0.001
Type of production	Beef	234	14	6.0%	Reference			
	Dairy	1782	112	6.3%	1.215 (0.642–2.302)	0.550	1.283 (0.681–2.415)	0.441
	Mixed	648	89	13.7%	1.744 (0.919–3.310)	0.089	1.780 (0.939–3.375)	0.077
Vaccination	No	2324	133	5.7%	Reference			
	Yes	261	71	27.2%	3.083 (2.129–4.463)	<0.001	1.895 (1.375–2.612)	<0.001
Veterinary control	No	1922	128	6.7%	Reference			
	Yes	751	84	11.2%	1.183 (0.840–1.67)	0.336	–	
Abortions	No	1907	107	5.6%	Reference			
	Yes	730	106	14.5%	1.862 (1.346–2.575)	<0.001	3.130 (2.172–4.509)	<0.001
Reproduction system	Insemination	282	36	13.6%	Reference			
	Mixed	301	25	3.4%	0.548 (0.291–1.033)	0.063	–	
	Natural breeding	2040	155	9.3%	0.757 (0.474–1.210)	0.245	–	

**Figure A1:**
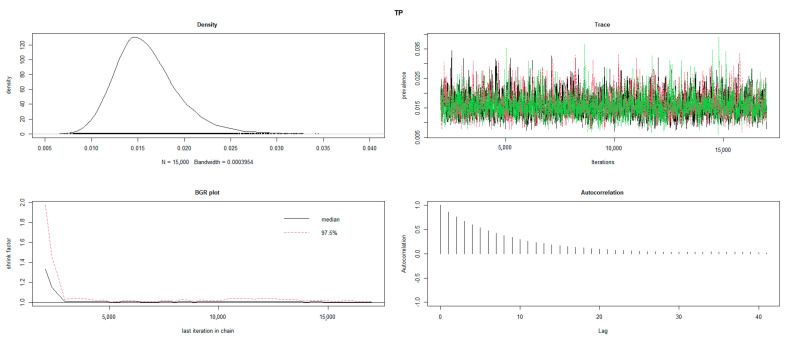
Density, trace, BGR plot, and autocorrelation for the true prevalence in Ecuador.

## Data Availability

The data that support the findings of this study are available from the corresponding author upon reasonable request.

## Appendix A

**Table A1:** Prior information used for sensitivity and specificity of RB and SAT and prevalence in the Coastal Region, Northern Highlands, Southern Highlands, and Amazon Region used in Models 1 and 2.

Region	MODEL 1			MODEL 2			REFERENCES
	Parameter	Value	Distribution	Parameter	Value	Distribution	
Coastal Region	theta [1]	4.0%	Beta = (9, 193)	TP	4%	Beta = (9, 193)	[3]
	theta [2]	66.4–96.0%	Beta = (16, 6)	SE [1]	66.4–96.0%	Beta = (16, 6)	[37]
	theta [3]	71.0–99.0%	Uniform = (0.71, 0.99)	SP [1]	71.0–99.0%	Uniform = (0.71, 0.99)	[37]
	theta [4]	NI	Uniform = (0.00, 1.00)	SE [2]	50.0–99.0%	Uniform = (0.55, 0.99)	[38]
	theta [5]	NI	Uniform = (0.00, 1.00)	SP [2]	90.0–100.0%	Uniform = (0.90, 1.0)	[38]
	theta [6]	NI	Uniform = (0.00, 1.00)	a [1]	NI	Uniform = (0.00, 0.25)	-
	theta [7]	NI	Uniform = (0.00, 1.00)	b [1]	NI	Uniform = (−0.25, 0.25)	-
Northern Highlands	theta [1]	2.5%	Beta = (5, 197)	TP	2.5%	Beta = (5, 197)	[3]
	theta [2]	66.4–96.0%	Beta = (16, 6)	SE [1]	66.4–96.0%	Beta = (16, 6)	[37]
	theta [3]	71–99%	Uniform = (0.71, 0.99)	SP [1]	71.0–99.0%	Uniform = (0.71, 0.99)	[37]
	theta [4]	NI	Uniform = (0.70, 1.00)	SE [2]	50.0–99.0%	Uniform = (0.55, 0.99)	[38]
	theta [5]	NI	Uniform = (0.00, 1.00)	SP [2]	90.0–100.0%	Uniform = (0.90, 1.00)	[38]
	theta [6]	NI	Uniform = (0.00, 1.00)	a [1]	NI	Uniform = (0.00, 0.25)	-
	theta [7]	NI	Uniform = (0.00, 1.00)	b [1]	NI	Uniform = (−0.25, 0.25)	-
Southern Highlands	theta [1]	0.5%	Beta = (1, 201)	TP	0.5%	Beta = (1, 201)	[3]
	theta [2]	66.4–96.0%	Beta = (16, 6)	SE [1]	66.4–96.0%	Beta = (16, 6)	[37]
	theta [3]	71.0–99.0%	Beta = (20, 2)	SP [1]	71.0–99.0%	Beta = (20, 2)	[37]
	theta [4]	NI	Uniform = (0.40, 1.00)	SE [2]	30.0%–90.0%	Uniform = (0.30, 0.90)	Supposed
	theta [5]	NI	Uniform = (0.00, 0.60)	SP [2]	90.0–100.0%	Uniform = (0.90, 1.00)	[38]
	theta [6]	NI	Uniform = (0.00, 1.00)	a [1]	NI	Uniform = (0.00, 0.25)	-
	theta [7]	NI	Uniform = (0.00, 0.60)	b [1]	NI	Uniform = (−0.25, 0.25)	-
Amazon Region	theta [1]	1%	Beta = (2, 200)	TP	1%	Beta = (2, 200)	[57]
	theta [2]	66.4–96.0%	Beta = (18, 4)	SE [1]	66.4–96.0%	Beta = (18, 4)	[37]
	theta [3]	71.0–99.0%	Beta = (21, 1)	SP [1]	71.0–99.0%	Beta = (21, 1)	[37]
	theta [4]	NI	Uniform = (0.80, 1.00)	SE [2]	50.0–99.0%	Uniform = (0.55, 0.99)	[38]
	theta [5]	NI	Uniform = (0.00, 0.60)	SP [2]	90.0–100.0%	Uniform = (0.90, 1.00)	[38]
	theta [6]	NI	Uniform = (0.50, 1.00)	a [1]	NI	Uniform = (0.00, 0.25)	-
	theta [7]	NI	Uniform = (0.00, 0.60)	b [1]	NI	Uniform = (−0.25, 0.25)	-

Legend: NI = non-informative.

**Table A2:** Prior information used for sensitivity and specificity of RB and SAT and prevalence on farms with and without vaccination in Ecuador used in Models 1.

REGION	MODEL 1			REFERENCES
	Parameter	Value	Distribution	
With and without vaccination	theta [1]	3%	Beta = (7, 195)	[3]
	theta [2]	66.4–96.0%	Beta = (17, 5)	[37]
	theta [3]	71.0–99.0%	Beta = (20, 2)	[37]
	theta [4]	NI	Uniform = (0.00, 1.00)	-
	theta [5]	NI	Uniform = (0.00, 1.00)	-
	theta [6]	NI	Uniform = (0.00, 1.00)	-
	theta [7]	NI	Uniform = (0.00, 1.00)	-

Legend: NI = non-informative.

**Table A3:** Posterior means and 95% credibility intervals (CrI) of the true prevalence and test parameters obtained from two different models in the Coastal Region, Northern Highlands, Southern Highlands, and Amazon Region.

Region	Parameter	Model 1 (%)	Model 2 (%)
		Mean (CrI)	Mean (CrI)
Coastal Region	True prevalence	2.5% (1.3–3.8%)	2.5% (1.5–3.5%)
	$Se_{T1}$	72.4 (51.4–89.5%)	72.6% (58.2–87.5%)
	$Sp_{T1}$	98.5 (97.7–99.0%)	98.5% (97.8–99.0%)
	$Se_{T2}$	69.6 (47.8–88.6%)	70.1% (56.3–86.9%)
	$Sp_{T2}$	98.6 (97.7–99.1%)	98.6% (97.9–99.1%)
	cov_a	0.150 (0.043–0.226)	0.157 (0.060–0.223)
	cov_b	0.014 (0.010–0.023)	0.013 (0.008–0.020)
	BGR	1.00	1.01
	Bayes-*p*	0.52	1.00
	DIC	20.07	36.72
Northern Highlands	True prevalence	1.0% (0.4–1.7%)	1.0% (0.4–1.6%)
	$Se_{T1}$	73.2% (53.2–89.2%)	72.6% (57.1–87.2%)
	$Sp_{T1}$	98.7% (98.2–99.0%)	98.7% (98.2–99.0%)
	$Se_{T2}$	70.8% (48.5–91.2%)	70.4% (56.1–89.6%)
	$Sp_{T2}$	98.7% (98.3–99.1%)	98.7% (98.3–99.1%)
	cov_a	0.121 (0.005–0.215)	0.128 (0.019–0.214)
	cov_b	0.013 (0.010–0.018)	0.012 (0.009–0.016)
	BGR	1.0009	1.0495
	Bayes-*p*	0.54	1.00
	DIC	17.81	32.170
Southern Highlands	Real prevalence	0.1% (0.0–0.3%)	0.1% (0.0–0.2%)
	$Se_{T1}$	73.0% (53.2–88.8%)	71.7% (52.5–87.2%)
	$Sp_{T1}$	99.8% (99.6–99.9%)	99.8% (99.7–99.9%)
	$Se_{T2}$	59.4% (33.9–85.5%)	59.7% (32.2–86.7%)
	$Sp_{T2}$	99.8% (99.6–99.9%)	99.8% (99.7–99.9%)
	cov_a	0.028 (−0.134–0.176)	0.088 (0.005–0.198)
	cov_b	0.002 (0.000–0.004)	0.002 (0.001–0.003)
	BGR	1.00	1.03
	Bayes-*p*	0.66	1.00
	DIC	11.36	18.41
Amazon Region	Real prevalence	0.8% (0.1–1.8%)	0.5% (0.1–1.4%)
	$Se_{T1}$	81.8% (64.0–94.5%)	78.8% (61.0–92.4%)
	$Sp_{T1}$	99.4% (98.5–100.0%)	99.1% (98.4–99.9%)
	$Se_{T2}$	81.0% (64.2–93.8%)	80.3% (58.2–96.7%)
	$Sp_{T2}$	99.2% (98.2–99.8%)	98.9% (98.1–99.7%)
	cov_a	0.060 (0.020–0.109)	0.073 (0.003–0.179)
	cov_b	0.006 (0.000–0.015)	0.008 (0.001–0.015)
	BGR	1.0002	1.0081
	Bayes-*p*	0.66	1.00
	DIC	11.87	20.27

Legend: $Se_{T1}\;$= sensibility of Rose Bengal test; $Sp_{T1}$ = specificity of Rose Bengal test; $Se_{T2}$ = sensibility of SAT-EDTA test; $Sp_{T2}$ = specificity of SAT-EDTA test; cov_a = relationship: RB and SAT-EDTA/disease; cov_b = relationship: RB and SAT-EDTA/absence of disease; BGR = multivariate Brooks–Gelman–Rubin (BGR) statistic; Bayes-*p* = Bayesian *p*-values; DIC = deviance information criterion.
